# Mortality trends in Australian Aboriginal peoples and New Zealand Māori

**DOI:** 10.1186/s12963-017-0140-6

**Published:** 2017-07-04

**Authors:** Bronwen Phillips, John Daniels, Alistair Woodward, Tony Blakely, Richard Taylor, Stephen Morrell

**Affiliations:** 1 School of Public Health and Community Medicine (SPHCM), Faculty of Medicine, University of New South Wales (UNSW), Kensington (Main) Campus, Samuels Building, Level 2, Room 223, Botany St, Gate 11, Randwick (Sydney), NSW 2052 Australia; 2 University of Wollongong, Wollongong, NSW Australia; 3 School of Population Health, University of Auckland, Auckland, New Zealand; 4 Department of Public Health, University of Otago, Wellington, New Zealand

**Keywords:** Indigenous, Māori, Life expectancy, Mortality, Adult mortality, Australia, New Zealand

## Abstract

**Background:**

The health status of Indigenous populations of Australia and New Zealand (NZ) Māori manifests as life expectancies substantially lower than the total population. Accurate assessment of time trends in mortality and life expectancy allows evaluation of progress in reduction of health inequalities compared to the national or non-Indigenous population.

**Methods:**

Age-specific mortality and life expectancy (at birth) (LE) for Indigenous populations (Australia from 1990 and NZ from 1950); and all Australia and non-Māori NZ (from 1890), males (M) and females (F), were obtained from published sources and national statistical agency reports. Period trends were assessed for credible estimates of Indigenous LE, and the LE gap compared to the total population for Australia, and non-Māori for NZ. Period trends in premature adult mortality, as cumulative probability of dying over 15–59 years, were assessed similarly. The relative contribution of differences in age-specific mortality to the LE gap between Indigenous and the all-Australia population, and the non-Māori NZ, was estimated for each country by sex for the most recent period: 2010–2012 for Australia, 2012–2014 for NZ.

**Results:**

LE increased for all populations, although LE gaps between Indigenous and all Australia showed little change over time. LE gaps between NZ Māori and non-Māori increased significantly from the early 1980s to the mid-1990s, and since then have fallen again. Recent LE gaps in Australia (M 12.5; F 12.0 years in 2010–2012) were larger than in NZ (M 7.3; F 6.8 years in 2012–2014). Premature adult mortality (15–59 years) improved for all populations, but mortality ratios show little change since 2000, with Indigenous at 3½-4 times that of all Australians, and Māori 2–3 times that of non-Māori. Using decomposition analysis, the age interval contributing most strongly to differences in LE between Indigenous and all Australia was 35–59 years, but between Māori and non-Māori it was 60–74 years.

**Conclusion:**

In Australia and NZ, Indigenous LE and adult mortality are improving in absolute terms, but not relative to the entire or non-Indigenous populations, causing gaps in life expectancy to persist.

## Background

Recent life expectancies at birth (LE) reported by the Australian Bureau of Statistics (ABS) of 80 years for males (M) and 84 years for females (F) (2010–2012) [1], and by Statistics New Zealand (Stats NZ) of 79 and 83 years [2] for males and females (2012–2014), come from accurate death registration and population denominators from censuses. The gaps in LE for Indigenous compared with all Australia were estimated as 12.5 and 12.0 years for males and females by ABS [1], and for NZ Māori compared to non-Māori were 7.3 and 6.8 years for males and females by Stats NZ [2, 3].

In Australia, reported Indigenous infant mortality rates (IMR) declined over recent decades, from 21.9 in 1991 [4] to 6.4 in 2012 [5] per 1000 live births. In New Zealand, IMR declined from 13.8 in 1990 to 5.9 by 2012–2014 [2, 3]. Substantial persistent adult mortality from non-communicable diseases has been flagged as a critical public health and social issue in Indigenous populations in both Australia and New Zealand (ANZ) [1–3, 6–8].

Comparisons between Indigenous and non-Indigenous population counts and deaths in ANZ are problematic due to under-identification, under-enumeration, and changing propensities to identify as Indigenous in ANZ [1, 7, 9, 10]. Under-identification of Indigenous peoples in population censuses and death registries is common across several countries colonized by Europeans, such as Canada and the United States (US); the net result, if under-identification is greater in mortality than census data, is under-estimated mortality rates and over-estimated LE (numerator-denominator incongruity) [10–15].

In Australia, the ABS adjusted census data for Indigenous under-identification for the 1991–1996 [16] and 1996–2001 periods [17]. The indirect methods used then to estimate Indigenous mortality consequently have been considered to be less credible, over-estimating mortality and under-estimating LE, and have since been discarded [18, 19]. Subsequent data linkage of death registries and census records to improve Indigenous identification enabled direct calculation of mortality rates for 2005–2007 and 2010–2012 [1, 20], although these rates are considered to be under-estimates, with LE consequently over-estimated [1, 19, 20].

In NZ, under-identification of Māori population counts and deaths led to under-estimation of mortality and over-estimation of LE during 1980–1999 [7, 8, 21, 22]. The New Zealand Census-Mortality Study (NZCMS) enhanced ethnic identification through data linkage between death registrations and census records resulting in more realistic (and higher) mortality rates for Māori up to 1996 [7, 22]. Studies from other countries, such as Canada [23] and the US [11] have also used data linkage to improve Indigenous identification and attain more realistic mortality and LE estimates [11, 23].

LE alone, a common population health indicator [24], gives little insight into mortality profiles of populations, whereas age-specific mortality can identify age ranges contributing most to premature mortality and LE differences. Further, data on mortality trends are more informative than cross-sectional “snapshots”, and comparison of Indigenous minorities with total or non-Indigenous populations is important to quantify the mortality and LE “gap”.

This paper compares LE and adult all-cause mortality rates by sex for the Indigenous and entire populations of Australia, and between Māori and non-Māori of NZ, from the late nineteenth century to 2012–2014, where data are available. Assessments are made of trends in the gap between Indigenous and total or non-Indigenous LE, also in relation to changing estimation methods; and estimates are made of the contributions of age-specific mortality to life expectancy differentials for the most recent period, 2012–2014.

## Methods

Mortality in Indigenous Australians, who are estimated to comprise 3% of the national population (in 2014) [25], is compared with all Australians, the conventional comparison by statistical agencies [25, 26]. Due to the proportionately larger NZ Māori population, which comprises 15% of the total NZ population, Māori death rates affect all NZ death data, hence the NZ convention, followed here, compares Māori with non-Māori [2].

Period life tables are constructed from age-specific death rates to derive life expectancies [27]. Direct methods are used to calculate mortality rates from reported deaths and population counts (which may be adjusted or enhanced) over a particular period [12, 13]. Indirect methods estimate survivorship and mortality based on calculations and assumptions from changes in available aggregate data on population and deaths, or reported survivorship of relatives [12, 13, 28], covered in detail in Phillips et al. 2014 [19].

***Life expectancies at birth*** (LE) for all Australia were obtained from life tables published by the ABS [29] based on registered births and deaths. LE for Indigenous Australian populations [1, 16, 17, 20] were derived from mortality rates calculated through indirect methods by Preston-Hill for 1991–1996 [16], and Bhat for 1996–2001 [17]. A direct method for 2005–2007 [20] was employed by the ABS following enhancement of Indigenous designation through death and census record linkage. Two variants of this direct method were used for 2010–2012 [1], and the lower LE estimates are included in this analysis as they are affected less by under-enumeration of Indigenous decedents. Indigenous LE was also estimated by Hill et al. (2007) [30] based on reported deaths and populations from ABS, using the General Growth Balance (GGB) (indirect) method for 1991–1996 and 1996–2001 [30]. LE data from life tables based on age-specific mortality of a known urban Aboriginal cohort at an Aboriginal Community Controlled Health Service (ACCHS) in Sydney for 1995–1999, 2000–2004 and 2005–2009 are also included [31]. Calculation of differences in Indigenous versus all-Australia LE and cumulative probability of adult mortality in the Australian data use only those estimates considered credible. Weighing up the multiple sources of Australian Indigenous mortality estimation, we censored the estimates derived indirectly by Preston-Hill [16] and Bhat [17] as recommended by the ABS [18]. A detailed review of these various sources and estimation methods is beyond the scope of this paper and is published elsewhere [19].

Life tables published by Cheung (1999) [32] for NZ non-Māori, by Pool (1983, 1985) [33, 34] for Māori populations prior to 1950, and subsequently by Statistics NZ for NZ non-Māori and Māori populations [2, 3, 35–38], calculated by direct methods from deaths and populations, were used for this study. Following data linkage of death registrations and census records through the New Zealand Cohort-Mortality Study (NZCMS) [7] to improve Māori identification, Māori and non-Māori deaths were recalculated for 1980–1999 by Statistics NZ [7], and these are used in estimating LE and adult mortality differences. LE estimates are plotted, where available, for ANZ, by sex, from 1881–1890 to 2010–2012.

***Adult mortality*** was summarized as the cumulative probability of dying between ages 15 and 59 years (period method) designated as _*45*_ *q*_*15*_, the traditional demographic adult mortality range [24]. As for LE, this was calculated by Hill et al. (2007) for the Australian Indigenous population for 1991–1996 and 1996–2001 using the indirect GGB method [30]. For the remaining all-Australia and Indigenous population estimates, adult mortality was calculated from the abridged life tables published by the ABS, using _*45*_ *q*_*15*_ *= [1-Π(*_*n*_ *p*_*x*_ )]*1000 where _*n*_ *p*_*x*_ is the probability of surviving in a given age interval of *n* years, *x* the lower limit of each age range. Results were plotted at period mid-points, where available, from 1885 to 2011, by sex. Adult mortality was not directly re-calculable from NZCMS results [7], as complete life tables were unavailable. Thus, age-specific mortality rates from original NZ Stats life tables were adjusted for the four time periods between 1981 and 1999 using age-specific Māori adjustment ratios by Ajwani et al. (2003) [39] and _*45*_ *q*_*15*_ calculated as above.

Indigenous adult mortality rate ratios were calculated by dividing the Indigenous cumulated probability of dying between ages 15 to 59 years by the equivalent for the all-Australia, or the non-Māori population, and plotted by sex on a log scale from 1981 to 2012–2014 where available.

To show the main contributions to differences between Indigenous and non-Indigenous mortality, ***age- and sex-specific death rates*** were plotted for Indigenous and non-Indigenous populations for Australia and NZ for the most recent period available: 2008–2012 for Australia, calculated by the Australian Institute of Health and Welfare (AIHW) from ABS data [25]; and for 2012–2014 for NZ, from Statistics NZ [2, 3].

The ***contributions of mortality in each age interval to differences in LE*** between Indigenous and all Australia, and between Māori and non-Māori, were calculated according to a decomposition method by Arriaga (1984) [40] converted and described as “Arriaga’s Approach III” by Murthy Ponnapalli [41]. Results are plotted by percentage contribution to overall difference in LE, by sex, for Australia 2010–2012 and NZ 2012–2014.

## Results

**Fig. 1:**
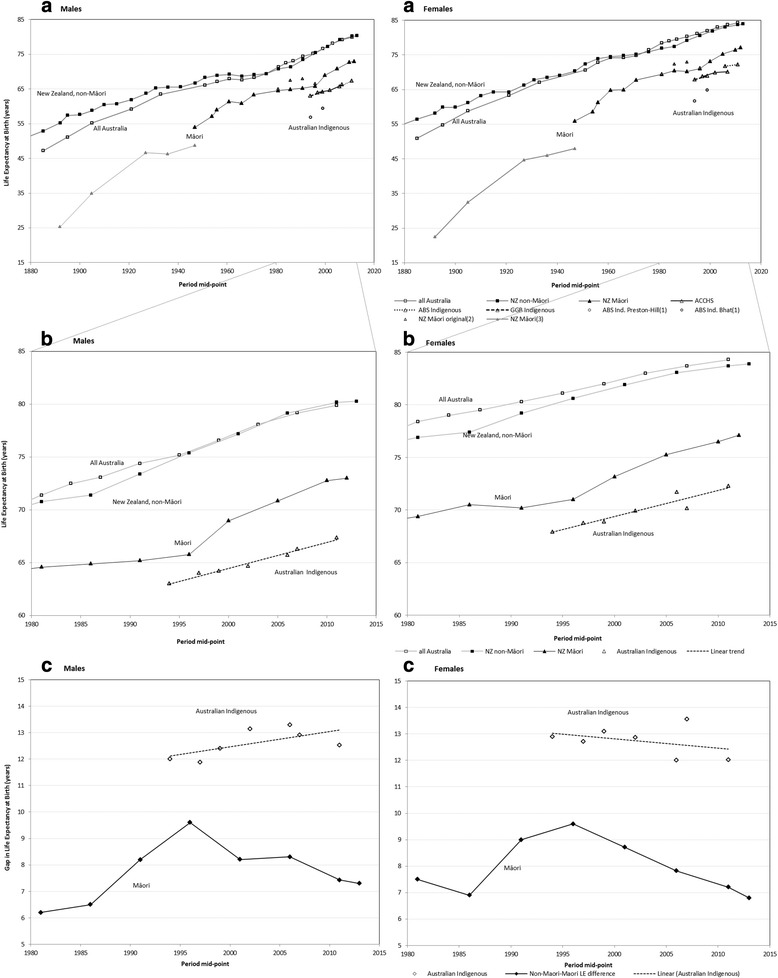
Life expectancy at birth for Indigenous and all Australians, and Māori and non-Māori New Zealanders, 1881–1890 to 2012–2014. **a**. Life expectancy at birth for specified populations, where available, from 1881 to 2014. **b**. Life expectancy at birth for specified populations, where available, from 1980 to 2014. **c**. Gaps in life expectancy at birth between specified populations, where available, from 1950 to 2014. (1) ABS Indigenous estimates no longer considered credible, circles (ABS Preston-Hill method [16], and ABS Bhat method [17], indirect methods). (2) Original NZ Māori estimates for 1981–1996 in light-shaded triangles, revised results in dark triangles with line. (3) NZ Māori estimates by Pool et al. 1891–1946 in light-shaded triangles. (4) Including revised 1981–96 NZ Māori estimates. ABS Indigenous from ABS (direct method) [1, 60]. ACCHS (Aboriginal Community Controlled Health Service): life tables based on mortality rates in a linked cohort (direct) [31]. GGB Indigenous (General Growth Method) from Hill et al. (2007) (indirect method) [30]. NZ Māori and non-Māori data from Cheung (1999) [32] and Statistics NZ [2, 3, 61] with revised NZ Māori data from Ajwani et al. (2003) [7] (direct methods)

***Overall life expectancy*** reported in ANZ shows a steady increase from the 1880s from around 50 years in Australia and 55 years in NZ, which slows from the 1930s, with plateaux for males and females in the 1960s–1970s (Fig. 1, a & b). LE improvement resumes from 1980 to reach 79.9 and 84.3 years for all-Australia males and females in 2010–2012, and 80.3 and 83.9 years for non-Māori NZ males and females in 2012–2014.

Three sources of mortality data for Indigenous Australians, employing different methodologies, provide similar LE estimates with a modest increase over the last two decades (Fig. 1, b) [1]. Despite Australian Indigenous LE improvements, when compared with the all Australia LE, the absolute gaps indicate an overall trend rise in males from ~12 to ~13 years and a slight trend decline in females, from ~13 to ~12.5 years (Fig. 1, c).

NZ Māori LE rose from 25.3 years for males and 22.5 years for females in 1891, to 46.3 and 46.0 years for males and females in 1946, as estimated by Pool. From the later Statistics NZ series, LE for NZ Māori increased from 54.1 years in 1950–1952 for males and 55.9 years for females until the 1960s, where the plateaux for males and females occur later than in the non-Māori (Fig. 1, a). LE improvement resumed in the 1970s then paused, for both sexes, between 1980 and 1984 and 1995–1999 (based on revised Māori data [7]), followed by LE increases from 2000 (Fig. 1, a & b). The LE gap between the non-Māori and Māori populations was 6.5 years for males and 6.9 for females in 1985–1987 (based on revised data by NZCMS) [7]; this increased to 7.3 years for males and decreased slightly to 6.8 for females in 2012–2014 (Fig. 1, c).

**Fig. 2:**
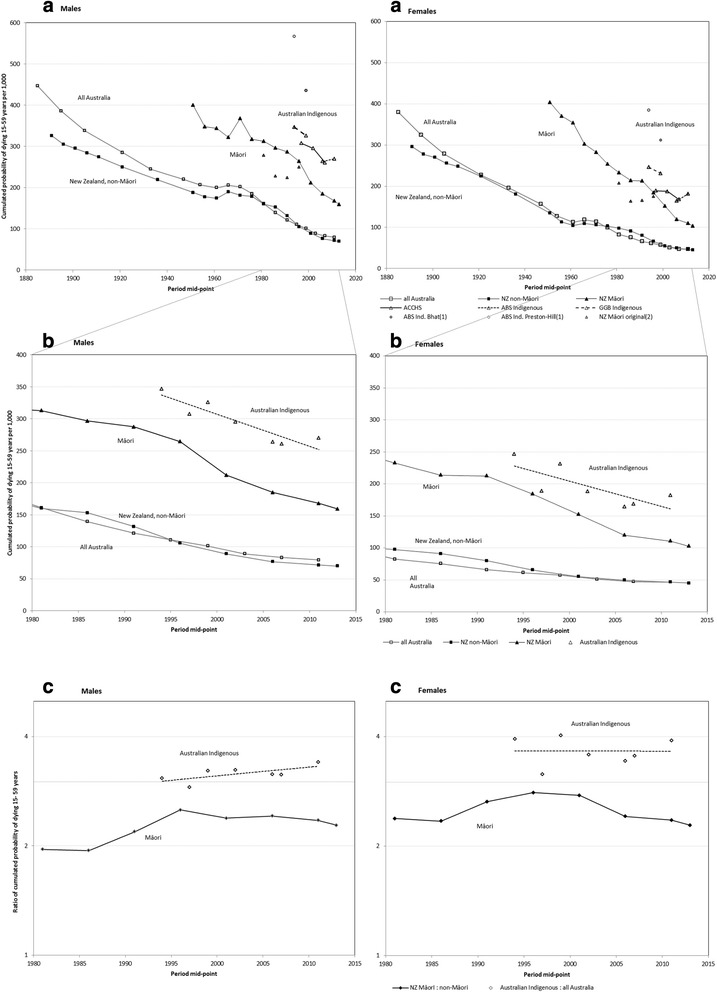
Adult (15–59 years) cumulative mortality for Indigenous and all Australians, and Māori and non-Māori New Zealanders, 1881–1890 to 2012–2014. **a**. 15–59 years Adult mortality, where available, 1881–2014. **b**. 15–59 years Adult mortality, where available, 1980–2014. **c**. Adult mortality rate ratios, 1980–2014. Adult mortality rates calculated using the probability of dying at ages 15–59 years (_15_q_45_ = [1-Π(_n_p_x_)]*1000) derived from: published life tables for ABS, from ABS (direct method, 1) [1, 60]; ABS (Preston-Hill method) [16]; ABS (Bhat method) [17]; authors’ calculations of adult mortality from Aboriginal Community Controlled Health Service (ACCHS) life tables; GGB Indigenous (General Growth Balance, Indigenous) adult mortality rates calculated and published by Hill et al. (2007) [30]; NZ non-Māori and Māori from Statistics NZ [2, 3, 37, 38, 61, 62]. (1) ABS Australian Indigenous estimates from methodologies no longer considered credible appear in circles. (2) Original NZ Māori data 1981–1996 in grey triangles; revised results in dark triangles, joined by lines

***The cumulated probability of adult mortality*** (15–59 years) decreased from 45% in all-Australia males and 38% in females in 1881–1890 until the 1960s–1970s when it leveled out to around 20% in males and 12% in females (Fig. 2, a). Further declines followed from the 1980s, to 8% for males and 5% for females by 2010–2012 (Fig. 2, a & b). NZ non-Māori adult cumulated mortality decreased from 33% (males) and 30% (females) in the 1890s [37] until the 1960s–1970s when both sexes followed a similar leveling pattern to Australia, then resumed its decline from 1980 to reach 7% for males and 4.5% for females by 2012–2014.

From available credible data, Australian Indigenous cumulated adult mortality declined from the 1990s to 2011, but there was no fall relative to all-Australia adult mortality (Fig. 2, a & b). Ratios of cumulated adult mortality were 3.14 for males and 3.43 for females in 2005–2007, and increased to 3.40 for males and to 3.90 for females in 2010–2012 (Fig. 2, c). The trend for males appears to be increasing while that for females appears steady. NZ Māori males show a decrease in adult mortality from 1950 (Fig. 2, a) which becomes steeper in the early twenty-first century. Māori female adult mortality declines from the 1950s, plateaus during the 1980s, then resumes the decline from the 1990s. In males, ratios of cumulated adult mortality of ~2 in 1980 rose to ~2.5 in the 1990s and declined slowly to 2.3 by 2012–2014); in females the mortality ratio decreased from ~2.5 in 1980 to 2.8 in the 1990s and declined more rapidly than males over 2000–05 to also reach ~2.3 by 2012–2014 (Fig. 2, c).

**Fig. 3:**
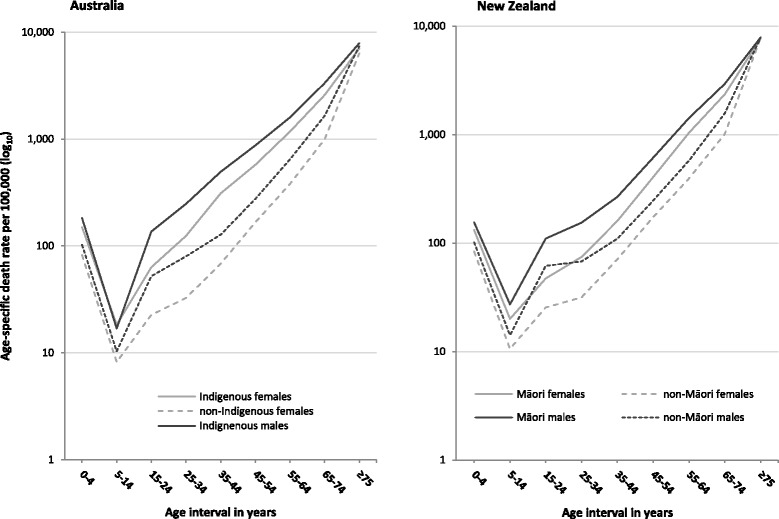
Age-specific death rates for Indigenous and non-Indigenous Australians, 2008–2012, and Māori and non-Māori New Zealanders, 2012–2014. AIHW from ABS data using a direct method, 2008–2012 [25], the latest comparable data available for the Indigenous and non-Indigenous populations of Australia. Statistics NZ for NZ data with age intervals aggregated to match Australia, 2012–2014 [2], the latest comparable data available for Māori and non-Māori populations of NZ

***Age-specific death rates*** for Indigenous and non-Indigenous populations of Australia (2008–2012) and NZ (2012–2014), by sex (plotted as logarithms), reveal higher death rates for Indigenous populations in all age intervals for both countries and sexes (Figure 3). Differences between Indigenous and non-Indigenous Australian populations were greater between ages 15 and 64 years, with the largest difference in the 35–44 years age interval. For NZ, the greatest mortality gaps between Māori and non-Māori occur in the 35–64 years age groups for males and in the 55–64 years age group for females.

**Fig. 4:**
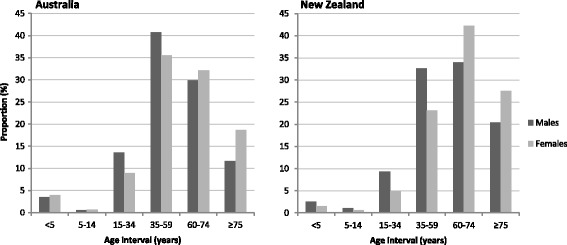
Age-specific mortality contributions to differences in life expectancy between Indigenous and all Australians (2010–2012), and Māori and non-Māori New Zealanders (2012–2014). Proportion (%) of LE difference contributed by each age interval. Differences in life expectancy using Arriaga’s decomposition method [40] converted to “Arriaga’s Approach III” by Murthy Ponnapalli [41]. From ABS life tables for Aboriginal and all Australia, 2010–2012, derived using a direct method [1, 5] and Statistics NZ life tables for Māori and non-Māori, 2012–2014 [2]

***Age-specific mortality contributions to LE differences*** in middle-aged 35–59 year adults contributed the most to LE differences between Indigenous and all Australia: 41% of the male difference and 36% of the female difference (Figure 4). In contrast, for Māori versus non-Māori, the 60–74 year age range contributed most to the LE difference, accounting for 34% of the difference in males, and 42% in females. Compared to Australia, the NZ 35–59 year age range contributed 33% in males and 23% in females to the LE difference.

## Discussion

By the first decade of the twenty-first century, Australian Indigenous LE at birth was estimated as 66–67 years for males and 70–72 years for females from a combination of: ABS estimation of mortality with enhancement of Indigenous designation through data linkage (although these estimates may be somewhat inflated from incomplete linkage) [1]; and ACCHS cohort data (Sydney). This produces a gap of approximately 13 years for each sex compared to all Australians [31]. For the 1990s, credible estimates of Australian Indigenous LE were 63–64 years for males and 68–70 years for females from the GGB method of Hill et al. [30]; and the ACCHS Sydney cohort. This produces a 12–13 year gap for each sex compared to all Australians [31]. Despite evidence of increased Indigenous Australian LE since the 1990s, the LE gap overall, compared with all Australians, has not diminished, with an increasing trend in males not quite negated by a less steeply declining trend in females.

From the first estimates in 1950–1952, LE for Māori converged initially with non-Māori, then widened during the 1990s, and has begun to close again to the most recent period, 2012–2014 [2]. There remains a gap of about 7 years in both sexes [2, 3] associated with rapid increases in non-Māori LE in the last decade [2, 3, 10, 36]. The LE in Indigenous Australians, compared to NZ Māori, is around 5 years less for both sexes, and the gap between Indigenous Australians and all Australians of 12–13 years is almost double that observed for New Zealand Māori and non-Māori.

The ratio of adult mortality in Māori to non-Māori was nearly 2½ times for men and women [36] whereas the ratio between Indigenous and all Australians was 3½ to 4 times for men and women. For the Indigenous population in Australia, the 35–59 year age range contributed most to the gap in life expectancy with all Australians, whereas for NZ Māori the 60–74 year age range contributed most to the gap in LE with NZ non-Māori.

### Data quality issues and variation in indigenous mortality estimation

Trends in LE for ANZ Indigenous populations are difficult to identify with confidence due to data quality issues including under-identification, under-enumeration, and changes in propensity to identify as Indigenous [7, 42, 43]. In Australia, estimates produced by the Preston-Hill [16] and Bhat [17] methods led to life expectancies in the 1990s of under 60 years for Indigenous males and under 65 years for Indigenous females. These were considerably lower than those derived by subsequent indirect approaches and the former methods have since been discarded. Hill et al. [30] used a generalized growth balance method which considered changes in propensity to identify as Indigenous as changes in census coverage; this approach estimated Indigenous LE during the 1990s as 63.5 years for males and 75.5 years for females (LE gap of 12 years for males and 13 years for females). More recent ABS estimates for 2005–2007 and 2010–2012 produced lower LE estimates based on enhancement of Indigenous status of deaths through linkage with census data than without such linkage. However, there is likely under-enumeration of deaths from incomplete Indigenous designation at death certification (leading to LE overestimates), and the ABS warns against period comparison because of possible numerator/denominator bias [44]. Madden et al. (2012) [45] noted that accuracy and consistency in ABS Indigenous LE and mortality analysis are different concepts. While the ABS prefers a consistent approach, this can be at the cost of accuracy, which may be improved, for example, by linkage of multiple datasets [45]. For the purposes of assessing changes over time within populations, consistency is more useful; for comparing different populations directly, accuracy is more important.

To a large extent both Indigenous population and mortality data quality in Australia makes direct Indigenous versus non-Indigenous comparisons fraught, due both to population enumeration of Indigenous status at the census and in death recording [19]. Until the late 1990s, Indigenous mortality was not reliably recorded outside of Western Australia, Northern Territory and South Australia, and trends in Indigenous self-designation at population censuses have not reflected natural population fluctuations (birth rates, etc.), but rather the propensity for change in Indigenous self-designation [19]. The secular variation in numerator-denominator incongruity is complex, and this is one reason why the ABS has not produced temporal Indigenous versus non-Indigenous mortality and LE estimates. However, as the proportion of the Indigenous to the all-Australian population is 3%, differences in mortality and LE estimates in relation to the whole population compared to the non-Indigenous population are very small and would bias any Australian Indigenous/non-Indigenous mortality or LE differentials slightly toward the null.

Increasing Aboriginal identification through data linkage may lead to further changes in Aboriginal mortality and morbidity measurements [9, 45, 46]. For example, from 2001 to 2012 the AIHW aimed to increase Indigenous identification by linking death registrations with three health databases (Residential Aged Care, the National Hospital Morbidity Database and the National Perinatal Data Collection) using an “ever-Indigenous” approach where an individual was considered Indigenous if this was indicated in any database [9, 46]. As a result, Indigenous mortality rates would have been revised upwards from identification of more Indigenous deaths.

Mortality in an urban Aboriginal cohort (75% living in Sydney postcodes) from 1995 to 2009 [31] was calculated with accurate designation of Indigenous status in the numerator and denominator. The ACCHS study included 24,035 people who first presented between 1980 and 2009, and made up 16% of the NSW Indigenous population at the 2006 Census. Linkage of this cohort to the National Death Index (NDI) showed 10% of the deaths were known to the health service, but could not be linked to the NDI, indicating that the national statistics very likely under-estimate Indigenous mortality. The LE and mortality estimates from the ACCHS source represent the first empirical estimates of Indigenous mortality from an overwhelmingly urban and substantial sample from the Australian Eastern seaboard. Moreover, the estimates from this source are congruent with credible estimates provided by Hill [30] and by the ABS [1].

NZ Māori LE estimates from 1950s–1970s may be inaccurate by up to 2 years [8], and as such inferences from comparing trends in this period with those in later periods need to be treated with caution. Another major recognized issue in NZ Māori mortality measurement occurred in 1981–1999, particularly 1980–1994, when the method of assigning ethnicity at time of death was deficient, leading to significant under-estimation of Māori mortality and consequent inflated Māori LE estimates. This Māori under-identification [7, 8] was corrected by the NZCMS study (1980–2006; 2006–2011) [7, 15, 21, 47] using undercount adjustment after probabilistic linkage of census and mortality records; the analysis also indicated that the Statistics NZ mortality estimates for 1995–1999 were approximately correct [7]. Re-analysis of trends revealed little increase in Māori LE over 1980–1999 and widening LE gaps with non-Māori over this period [7, 8, 10]. From 1980 to 1990 mortality improvement slowed for Māori males and stalled altogether for Māori females. Adult cumulated mortality ratios over 1950–2014 for Māori compared with non-Māori decreased in the 1960s–1980s, increased in the 1990s then decreased subsequently. Overall, New Zealand Māori mortality data are more reliable and extend for the best part of a century longer than corresponding Australian data, with time series and secular trends in these facing fewer issues than those for Australian Indigenous LE and mortality trends.

### Sources of differences in LE gaps

The most recent LE gaps estimated between all-Australian and Australian Indigenous populations were almost twice those between NZ non-Māori and Māori populations [1, 3]. The highest contribution to these LE gaps by age group was from the 35–59 year age range for Australia; and the older 60–74 year age range for NZ. In both countries, males showed greater levels of premature adult mortality at younger ages (15–34 and 35–59 years) than females. The higher 15–34 year contribution from Australian Indigenous males and females suggests external cause mortality playing a greater part than in Māori. Under 5-year mortality, less than 4% for both sexes and countries [1, 2], contributed far less to the recent LE gaps than adult mortality. Overall, the greater contributions at younger ages to the LE deficit in Indigenous Australians, compared to those in NZ Māori, imparts additional years of life lost to Indigenous Australians than if the LE deficit had a similar age composition to that of NZ Māori.

In the Northern Territory (NT) (Indigenous population: 64,005 at 2006 census - 12% of Indigenous Australians) [29], Indigenous LE increased from 52 years in 1967–1971 to 60 years in 2000–2004 for males, and from 54 to 68 years for females [48]. Compared to the all-Australia population, the major part of LE increase from 1967 to 1971 to 1984–1988 resulted from declines in infant mortality [48]. From 1984 to 1988 to 2000–2004, the improvement was due mainly to the decline in ≥65 years mortality [48]. Adults aged 15–64 years showed little change in mortality over this time [48]. Compared with the all-Australia population, the LE gap for NT Indigenous males increased from 16 to 18 years, but for females it decreased from 20 to 15 years [48]. In the NT 80% of the Indigenous population live in remote and very remote areas, compared with 25% of Indigenous people nationally [49]. Analysis of urban and regional Indigenous populations such as the Sydney ACCHS cohort [31] would be beneficial, although difficult given identification and registration issues in Australian jurisdictions outside NT and WA.

The rise in premature mortality from cardiovascular disease [50] had a profound effect on LE in ANZ over the 1950–70 period, moreso in males than females [50]. This period antedates any reliable data on Australian Indigenous mortality. NZ LE trends demonstrate a plateau from 1950 to 1970 for non-Māori (more pronounced for males), and a slowing down of LE increases in the Māori population that commenced simultaneously or a little later, and extended into the 1990s. However, results should be interpreted with caution, especially the exact magnitude of absolute Māori/non-Māori LE differences due to shortcomings in the records, including numerator/denominator bias varying over time [8, 35].

The plateaux in Māori mortality in the 1980s–1990s, and widening of the LE gap with the non-Māori population, were associated with dramatic social and macroeconomic changes [7] introduced by successive governments beginning in the 1970s [10, 51], frequently labeled “Rogernomics” after Lange Labour Government Minister of Finance Roger Douglas (1984–1988) [10]. These policies cut benefits and public services, led to large-scale job losses, reduced accessible housing for lower socioeconomic groups and restricted health services with the introduction of “user-pays” systems [7, 8, 10]. These changes were most damaging for economically-vulnerable groups including Māori and resulted in increased social and economic inequalities that contributed to widening health inequalities [7, 10].

Non-communicable diseases have contributed most heavily to premature mortality in Indigenous Australians and Māori in recent years [26, 35]. In 2013, Australian Indigenous mortality rates were highest for ischemic heart disease, diabetes, chronic lower respiratory disease, cerebrovascular disease, lung cancer and intentional self-harm; mortality from diabetes was six times than that seen in the non-Indigenous Australian population [26]. Assault was six times higher, cirrhosis and other liver diseases nearly five times more frequent, and cervical cancer three times higher as causes of death amongst Indigenous compared to the non-Indigenous population [26]. These leading causes of Indigenous deaths have been consistent since national data became available from the ABS in 2009, and since the 2003 Burden of Disease report was released in 2007 [6, 20]. By comparison, in 2009 Māori showed higher age-standardized mortality rates from diabetes, chronic rheumatic heart disease, lung, stomach and cervical cancers, chronic lower respiratory disease, transport accidents, hypertensive disease and assault [35]. Diabetes was reported as six times, and chronic rheumatic heart disease five times, more frequent in Māori than in the non-Māori population [35]. Moreover, updated analyses to 2011 from the NZCMS show the increasing contribution of diabetes and cancer to ethnic inequalities in mortality, as the cardiovascular epidemic declines [52].

### Indigenous mortality in other countries

Substantial adult mortality differentials between indigenous and non-indigenous populations in more-developed countries exist also in North America, and as in ANZ, indigenous deaths tend to be under-identified, with mortality under-estimated and LE over-estimated [11, 53, 54]. Large recent increases in American Indian/Alaskan Native (AI/AN) populations have resulted from changes in the propensity to identify as such in censuses [11]. Recent US National Death Index data linkage with Indian Health Service (IHS) registration in targeted regions (1990–2009) yielded higher mortality rates in all age groups for AI/AN compared with the European American population [53, 55, 56]. LE for AI/AN in 2007–2009 was estimated at 68.0 and 74.3 years for males and females, with gaps of 8.0 and 6.4 years compared with males and females of European descent [57]. Espey et al. (2014) [55] concluded that while declines in infant mortality were evident, the greatest disparities were in adult mortality, particularly those aged 24–44 years, influenced by diabetes, smoking, problem drinking and lack of access to health care [55].

The 1991–2001 Canadian Census mortality follow-up study that tracked mortality in a 15% sample of Canadian residents (1.5% identified as Aboriginal: Registered Indians of First Nations, *Métis* or Inuit), was an improvement on previous under-identified Canadian Aboriginal death data [23, 58, 59]. From the Aboriginal cohort aged 25 years or older at the 1991 Census, LE (at age 25 years, *e*_25_) gaps of 4.4 years for male and 6.3 for female were evident for Registered Indians compared with non-Aboriginals; and gaps of 3.3 and 5.5 years for *Métis* males and females were estimated. LE at 25 years (*e*_25_) for urban Aboriginal men and women were 4.7 and 6.5 years lower than urban non-Aboriginal men and women [23]. The mortality rate ratios for all Aboriginal groups (compared with the non-Aboriginal population) were highest in the 25–34 year age interval, followed by the 35–44 year age group [23].

Finally, it is difficult to estimate the degree of uncertainty associated with trends in ANZ Indigenous LE differences and mortality ratios. While there does appear to be discernible trends in these, absolute differences between, for example, Australian Indigenous and NZ Māori LEs, may be biased. Yet secular comparisons may not be seriously affected if the biases remain constant over time. Unfortunately, an examination of Australian Indigenous mortality estimates cannot assume even this. However, in our view careful selection of the most consistent and reliable estimates for comparison can provide a reasonable guide to the extent of secular changes in Indigenous life expectancy and life expectancy deficits.

## Conclusions

Indigenous Australian and NZ Māori have shown declines in adult mortality rates and increased LE over measurable time periods, but LE gaps are substantial and remain. Since the 1990s in Australia LE gaps between the Indigenous and the all-Australia populations appear not to have changed appreciably. This contrasts with the notable widening of the Māori LE gap during the 1980s and 1990s coinciding with the “Rogernomics” period in New Zealand. Although the NZ LE gap did not widen to the extent of that in Australia, the NZ LE decline does suggest the extent that changes to the social and economic environment can impact on the LE gap; and that the wider LE gap in Australia than NZ is a direct reflection of the substantially wider Indigenous/non-Indigenous economic disparity in Australia than NZ, despite otherwise similar levels of economic development in the two countries. Furthermore, similar to North America, most of the LE gap in ANZ originates from the adult age groups most affected by non-communicable disease mortality.
